# Next-Generation Sequencing: A Promising Tool for Vaccines and Other Biological Products

**DOI:** 10.3390/vaccines11030527

**Published:** 2023-02-23

**Authors:** Srinivas Nellimarla, Prasad Kesanakurti

**Affiliations:** Genomic Medicine Unit (GMU), Sanofi, 225 2nd Avenue, Waltham, MA 02451, USA

Next-generation sequencing (NGS), also known as high-throughput sequencing (HTS), is a commonly used term to represent a set of DNA sequencing technologies that have been in use for almost two decades. Unlike the Sanger sequencing method, which is a classical or first-generation sequencing technology, NGS methods simultaneously generate millions of sequencing reads in a single run within a relatively short span of time and at a lower cost [1]. NGS methods can be broadly classified into short-read and long-read sequencing technologies based on the read length of generated sequences. Short-read technologies can produce reads of 250–800 bases while long-read technologies can routinely generate reads longer than 10 kb. Both technologies have advantages and limitations. The capability of short-read technology to produce more accurate and deep sequencing data is limited by its poor ability to properly identify truncated fragments and chimeric sequences in therapeutic vectors and correctly sequence structural variants in genomes. These limitations can be overcome by long-read technologies that can sequence through these problematic regions although with less accuracy [2]. However, the combination of these two technologies is creating a wealth of genomic information. This incredible capacity of NGS methods to sequence large volumes of genetic material has revolutionized both basic and clinical research. The generation of substantial sequencing datasets by NGS provides opportunities to address new challenges in clinical areas, including infectious agents, vaccine development, and safety-related aspects of biologic therapeutic drug products, such as adventitious agent testing.

A crucial aspect of vaccine development is to understand the evolutionary trajectory of infectious agents in the most efficient way possible because infectious agents such as viruses tend to mutate at a constant and rapid rate, ultimately affecting the way in which they are transmitted. It is the speed and accuracy of the NGS that allows scientists to sequence multiple genomic variants of target viruses and develop potential vaccines [3]. Additionally, NGS can ensure that genetically modified regions of the vaccine virus genome remain as designed and, in this context, a recent study confirmed NGS to be suitable for vaccine lot release [4]. NGS has proven to be indispensable during the recent COVID-19 pandemic where the progress from pathogen discovery to novel vaccine development was made in record time. Recently, a comprehensive in silico analysis of several features of SARS-CoV-2 genomic sequence was performed in comparison with other members of the coronaviridae virus family to design an attenuated virus for vaccine development [5]. NGS is not only used in the early stages of vaccine development, but also to determine vaccine effectiveness by collecting and analyzing information about the vaccine’s interaction with the virus and the human body [6]. Similarly, a recent review documents the utilization and implementation of NGS technology in the identification of infectious disease outbreaks, genetic variants, transmission dynamics, spill-over events, and vaccine development [7]. Cancer vaccination is another clinical area that is considered a promising therapeutic strategy in treating solid tumors, and the wide accessibility of NGS has brought personalized cancer vaccines to the forefront of cancer research. Several studies have discussed the applications of NGS in the prediction of neoantigen targets for cancer vaccines and the design of personalized neoantigen RNA vaccines against cancer [8,9].

Safety is key to the development of any medicine. NGS has also emerged as an analytical tool in the realm of biologics to identify highly sensitive adventitious agents in the manufacturing process. Adventitious viral testing is a vital quality assessment method that guarantees the safety and preserves public confidence in the usage of vaccines and other biologicals. Current tests available for adventitious viral testing are time-consuming, heavily dependent on animal (in vivo) testing, and inaccurate [10]. Lengthy testing periods can be a concern for some gene therapy products that must be administered to patients shortly after production. Sample volume is another concern for some gene therapy products, particularly for viral vectors. Several studies have shown the ability of NGS technology either to replace or supplement existing testing methods [11,12]. Most importantly, a recent study published by the MIT Center for Biomedical Innovation collected and evaluated historical data via in vivo methods from 20 biopharmaceutical industry members to recommend NGS as a potential tool for replacing in vivo adventitious virus testing [13]. However, there are several challenges in the adoption of techniques such as NGS, including the risk associated with new platforms, resources needed, fear of regulator delays and hesitancy to share information. While these are valid reasons, the requests for using NGS are significantly increasing. A noteworthy development in this direction is the revised ICH Q5A (R2) guidelines [14] that allow NGS as a replacement method for current in vivo methods due to the breadth of viruses it detects and because of its ability to meet the intent of global objective to replace, remove, and refine the use of animals. Although NGS is recommended as a replacement method, validation studies still need to be initiated in large numbers. Such an initiative is necessary to establish, not just recommend, NGS as a better alternative to current viral safety methods.

In conclusion, the above collection of studies sheds light on the power of NGS in vaccine development and safety assessments. While it is still being used as a supplementary method for various quality testing procedures, with a continuous reduction in sequencing cost and increased support from regulators, as reflected in revised ICH Q5 (R2) guidelines, NGS is poised to become a replacement method for animal testing in pharmaceutics. Nevertheless, sustained support from both academia and industry in the form of collaboration and data generation is necessary to persuade regulatory agencies to accept NGS as a primary, rather than a supplementary, quality assessment tool.

## References

[1] Slatko B.E., Gardner A.F., Ausubel F.M. (2018). Overview of Next-Generation Sequencing Technologies. Curr. Protoc. Mol. Biol..

[2] Hu T., Chitnis N., Monos D., Dinh A. (2021). Next-generation sequencing technologies: An overview. Hum. Immunol..

[3] Nasereddin A., Berman H.G., Wolf D.G., Oiknine-Djian E., Adar S. (2022). Identification of SARS-CoV-2 Variants of concern using Amplicon Next-Generation Sequencing. Clin. Microbiol..

[4] Konz J.O., Schofield T., Carlyle S., Wahid R., Ansari A., Strating J.R.P.M., Te Yeh M., Manukyan H., Smits S.L., Tritama E. (2021). Evaluation and validation of next-generation sequencing to support lot release for a novel type 2 oral polio vaccine. Vaccine X.

[5] Kames J., Holcomb D.D., Kimchi O., DiCuccio M., Hamasaki-Katagiri N., Wang T., Komar A.A., Alexaki A., Kimchi-Sarfaty C. (2020). Sequence analysis of SARS-CoV-2 genome reveals features important for vaccine design. Sci. Rep..

[6] McNamara R.P., Caro-Vegas C., Landis J.T., Moorad R., Pluta L.J., Eason A.B., Thompson C., Bailey A., Villamor F.C.S., Lange P.T. (2020). High-density amplicon sequencing identifies community spread and ongoing evolution of SARS-CoV-2 in the Southern United States. Cell Rep..

[7] Quer J., Colomer-Castell S., Campos C., Andrés C., Piñana M., Cortese M.F., González-Sánchez A., Garcia-Cehic D., Ibáñez M., Pumarola T. (2022). Next-Generation Sequencing for Confronting Virus Pandemics. Viruses.

[8] Lancaster E.M., Jablons D., Kratz J.R. (2020). Applications of Next-Generation Sequencing in Neoantigen Prediction and Cancer Vaccine Development. Genet. Test Mol. Biomark..

[9] Alburquerque-González B., López-Abellán M.D., Luengo-Gil G., Montoro-García S., Conesa-Zamora P. (2022). Design of personalized neoantigen RNA vaccines against cancer based on next-generation sequencing data. Methods Mol. Biol..

[10] Gombold J., Karakasidis S., Niksa P., Podczasy J., Neumann K., Richardson J., Sane N., Johnson-Leva R., Randolph V., Sadoff J. (2014). Systematic evaluation of in vitro and in vivo adventitious virus assays for the detection of viral contamination of cell banks and biological products. Vaccine.

[11] Mallet L., Gisonni-Lex L. (2014). Need for new technologies for detection of adventitious agents in vaccines and other biological products. PDA J. Pharm. Sci. Technol..

[12] Ng S.H., Azizi A., Edamura K., Malott R.J., Charlebois R.L., Logvinoff C., Schreiber M., Mallet L., Gisonni-Lex L. (2014). Preliminary Evaluation of Next-Generation Sequencing Performance Relative to qPCR and In Vitro Cell Culture Tests for Human Cytomegalovirus. PDA J. Pharm. Sci. Technol..

[13] Barone P.W., Keumurian F.J., Neufeld C., Koenigsberg A., Kiss R., Leung J., Wiebe M., Ait-Belkacem R., Azimpour Tabrizi C., Barbirato C. (2023). Historical evaluation of the in vivo adventitious virus test and its potential for replacement with next generation sequencing (NGS). Biologicals.

[14] ICH Q5A (R2) Viral Safety Evaluation of Biotechnology Products Derived from Cell Lines of Human or Animal Origin. International Council for Harmonisation of Technical Requirements for Pharmaceuticals for Human Use. https://www.fda.gov/media/163115/download.

